# Isolation and antigenic characterization of calcareous corpuscle-associated proteins from Taenia solium cysticerci

**DOI:** 10.21203/rs.3.rs-9892193/v1

**Published:** 2026-07-27

**Authors:** Andrea Rivera, Adriana Paredes, Miguel Marzal, Patricia Sáenz, Mirko Zimic, Robert H Gilman, Hector H García

**Affiliations:** Universidad Ricardo Palma; Universidad Peruana Cayetano Heredia; Universidad Peruana Cayetano Heredia; Universidad Peruana Cayetano Heredia; Universidad Peruana Cayetano Heredia; Johns Hopkins University; Universidad Peruana Cayetano Heredia

**Keywords:** Taenia solium, Cysticercosis, Calcareous corpuscles, Parasite antigens, Monoclonal antibodies, Protein characterization

## Abstract

**Background:**

Calcareous corpuscles are mineralized structures characteristic of cestodes, including *Taenia solium*, yet their protein composition and antigenic properties remain poorly characterized. Existing studies have focused mainly on calcium-binding fractions, limiting understanding of the broader molecular content of these structures.

**Methods:**

A two-phase methodology was developed to isolate calcareous corpuscles from viable *T. solium* cysticerci obtained from naturally infected pigs and to recover their associated proteins. Corpuscles were isolated by mechanical homogenization and differential centrifugation, followed by controlled chemical dissolution. Protein recovery was assessed by Bradford assay and SDS-PAGE. Antigenic properties were evaluated using immunofluorescence and Western blot with a panel of monoclonal antibodies raised against whole cysticercus extract, vesicular fluid, and excretion–secretion antigens.

**Results:**

The optimized protocol consistently yielded intact calcareous corpuscles and enabled complete dissolution of the mineral matrix with sequential release of proteins. Electrophoretic analysis revealed a heterogeneous protein profile comprising at least 23 distinct protein bands. Several proteins were recurrently detected by multiple monoclonal antibodies, particularly bands around 66.9 kDa and in the low-molecular-weight range. Western blot analysis confirmed specific recognition of corpuscle-associated proteins by a subset of monoclonal antibodies.

**Conclusions:**

Calcareous corpuscle-enriched preparations yielded a diverse set of immunoreactive proteins. The methodology described here provides a reproducible framework for further proteomic characterization and may facilitate future proteomic, diagnostic, and immunological studies in cysticercosis and neurocysticercosis.

## 1. Introduction

Calcareous corpuscles are distinctive spherical mineralized structures found primarily in cestodes and also in some trematodes (von Brand et al., 1969; Señorale-Pose et al., 2008). These structures are composed primarily of calcium, magnesium, and phosphate salts embedded within an organic matrix (von Brand et al., 1960, 1967; von Brand and Nylen, 1970). Although their precise biological function remains incompletely understood, they have been associated with ion regulation, mineral storage, metabolic processes, and possibly detoxification mechanisms (Vargas-Parada and Laclette, 1999).

In cestodes, calcareous corpuscles have been morphologically described in several species (von Brand and Nylen, 1970; McCullough and Fairweather, 1987; Rodrigues et al., 1997; Yang, 2000; Zurabian et al., 2005), and isolation procedures have been reported in parasites such as *Spirometra erinaceid* (Park et al., 2005). In *Taenia solium*, the etiological agent of cysticercosis and neurocysticercosis, previous studies on calcareous corpuscles have focused mainly on calcium-binding protein fractions or specific biochemical components rather than evaluating the full spectrum of proteins potentially embedded within the mineral matrix (Zurabian et al., 2005).

Despite the structural characterization of calcareous corpuscles, their antigenic composition remains poorly defined. Understanding whether these mineralized bodies contain immunologically relevant proteins is particularly important in *T. solium*, where antigenic components of the cysticercus stage play a central role in host immune recognition, homeostasis and pathophysiology.

We hypothesized that calcareous corpuscles may harbor a selective subset of parasite proteins that retain antigenic properties. To test this hypothesis, we developed an isolation and dissolution protocol combining previously described methodologies with optimized washing and sulfamic acid–based solubilization steps to achieve complete corpuscle dissolution and protein release.

The proteins obtained were quantified and analyzed by SDS-PAGE and Western blot using monoclonal antibodies generated against whole cysticercus extract, vesicular fluid, or excretion/secretion antigens. Additionally, immunofluorescence assays were performed to confirm antigen localization in parasite tissue and isolated corpuscles. This study provides evidence that calcareous corpuscles of *T. solium* contain immunoreactive proteins, expanding the current understanding of their biological and immunological relevance.

## 2. Materials and methods

### 2.1 Parasite material

*T. solium* cysticerci were obtained from the skeletal muscle of three naturally infected pigs originating from an endemic region in the north of Peru, identified as part of a separate study on cysticercosis control (Garcia et al., 2016), reviewed and approved by the Institutional Animal Ethics Committee of the School of Veterinary Medicine, Universidad Nacional Mayor de San Marcos (approval number 2004–007).

Approximately 200 metacestodes were collected per animal, yielding a total of 600 cysticerci. To minimize variability related to individual hosts and to obtain a representative sample, cysticerci from the three animals were pooled prior to processing and handled as a single combined sample throughout the study. Parasites were transported to the laboratory in sterile phosphate-buffered saline (PBS, pH 7.4; Gibco-Invitrogen, Gaithersburg, MD) supplemented with broad-spectrum antibiotics (amoxicillin, streptomycin, and chloramphenicol) and protease inhibitors (1× PMSF) to preserve protein integrity.

Upon arrival, cysticerci were washed extensively in PBS and stored at − 70°C until processing. Prior to calcareous corpuscle isolation, samples were thawed at 4°C and processed immediately under cold conditions to minimize protein degradation.

### 2.2 Standardization of calcareous corpuscle isolation

The isolation protocol was developed based on previously described corpuscle enrichment procedures in cestodes (Zurabian et al., 2005), with modifications introduced to improve handling consistency, corpuscle enrichment, and downstream protein recovery.

Cysticerci were homogenized in phosphate buffer using a Polytron homogenizer (Kinematica PCU) at maximum intensity for 1–2 min at 4°C. Homogenization conditions were optimized to ensure adequate tissue disruption while preserving corpuscle integrity.

The homogenate was centrifuged at 12,000 g for 15 min at 4°C. A whitish pellet was consistently obtained and was considered morphologically indicative of corpuscle-enriched preparation. The supernatant containing soluble parasite components and cellular debris was discarded.

The pellet was subjected to three additional washing steps with phosphate buffer, each followed by centrifugation under identical conditions, to reduce contamination from non-corpuscular proteins.

### 2.3 Sequential dissolution and microscopic validation

Corpuscle dissolution was implemented drawing upon previously reported approaches for calcareous corpuscle solubilization (Park et al., 2005; Zurabian et al., 2005), with optimization of dissolution conditions and validation of complete structural disruption.

Purified corpuscles were treated with 0.1 M sulfamic acid solution (9.7%) adjusted to pH 1.2, at a ratio of 2:1 (v/v; acid solution volume relative to corpuscle pellet volume). Samples were vortexed at maximum speed for 1 min and centrifuged at 15,000 g for 5 min at 4°C.

Supernatants were collected after each dissolution step. Residual pellets were subjected to repeated sulfamic acid treatments until complete dissolution was confirmed by light microscopy. Microscopic evaluation was performed after each cycle to verify progressive structural disappearance of corpuscles. Each dissolution step yielded approximately 300 μL of supernatant per vial.

All collected supernatants were stored at − 70°C until further analysis.

### 2.4 Protein quantification

Protein concentration was determined from the supernatants obtained after sequential dissolution of calcareous corpuscles. Quantification was performed using the Bradford assay (Bio-Rad Protein Assay) in 96-well microplates.

Briefly, 20 μL of each sample were mixed with 200 μL of Bradford reagent diluted 1:50 in distilled water to minimize potential interference from the acidic conditions of the dissolution process. The mixture was incubated for 5 min at room temperature in the dark. Protein concentration was measured spectrophotometrically at 595 nm using a microplate reader (Molecular Devices Inc., Sunnyvale, CA).

A standard calibration curve was generated using bovine serum albumin (BSA) at concentrations of 0, 5, 10, 20, 40, 80, 100, and 200 μg/mL. Protein concentrations of corpuscle-derived fractions were calculated based on this standard curve.

### 2.5 SDS-PAGE analysis

Protein samples obtained from corpuscle dissolution supernatants were analyzed by SDS-PAGE using 10% polyacrylamide gels (stacking and resolving system).

Samples were prepared by mixing 35 μL of supernatant with 10 μL of 5× sample buffer and 5 μL glycerol. A total volume of 20 μL of each prepared sample was loaded per lane. A pre-stained molecular weight marker (Bio-Rad, 161–0318) was included for size estimation.

Electrophoresis was performed in a Mini-PROTEAN^®^ Tetra Vertical Electrophoresis Cell (Bio-Rad) using running buffer composed of 0.25 M Tris base, 2 M glycine, and 1% SDS. Gels were run at 80 V for 15 min during stacking and at 130 V for 2 h during resolving.

Protein bands were visualized using a silver staining kit (Silver Stain Plus, Bio-Rad) according to the manufacturer’s instructions.

### 2.6 Western blot analysis

Proteins separated by SDS-PAGE were electrotransferred onto nitrocellulose membranes using transfer buffer containing 25 mM Tris base, 192 mM glycine, and 20% methanol in Milli-Q water. Electrotransfer was performed at 70 V for 1 h.

Following transfer, nitrocellulose membranes were cut into strips and subjected to immunodetection. Membranes were blocked for 2 h at room temperature under constant agitation in blocking solution composed of PBS supplemented with 10% skim milk and 0.05% Tween-20 (PBS-T).

After blocking, membranes were washed three times with PBS-T (0.05% Tween-20) and incubated for 2 h at room temperature with a panel of in-house produced monoclonal antibodies generated against *T. solium* whole cysticercus extract, vesicular fluid, or excretion–secretion antigens (Tsang, Brand and Boyer, 1989). Monoclonal antibodies were used at a concentration of 10 μg/mL diluted in PBS-T (0.05% Tween-20).

Membranes were washed three times with PBS-T and subsequently incubated for 2 h at room temperature with goat anti-mouse secondary antibody conjugated to biotin and peroxidase (Goat anti-mouse biotin, ab6788) diluted 1:5000 in PBS-T containing 2% skim milk. Immunoreactive bands were visualized using streptavidin precipitating substrate according to the manufacturer’s instructions. Development was monitored for 10–15 min until bands became visible. All analyses were qualitative, and band intensity was not quantified.

### 2.7 Immunofluorescence assay

Eighteen in-house produced monoclonal antibodies generated against total *T. solium* cysticercus extract were available (Paredes et al., 2016) (see Supplementary Material, S1). To identify monoclonal antibodies recognizing calcareous corpuscle-associated antigens, a screening process was performed using immunofluorescence assays.

Two types of samples were evaluated: (i) parasite tissue sections and (ii) isolated calcareous corpuscles fixed onto glass slides.

#### 2.7.1 Immunofluorescence in parasite tissue sections

Immunofluorescence assays were initially performed on parasite tissue sections following a protocol previously described by Paredes et al (Paredes et al., 2016), with minor modifications. Briefly, cysticercus tissues obtained from naturally infected pigs were fixed in 4% formalin, paraffin-embedded, sectioned at 4 μm, and mounted onto poly-L-lysine–coated glass slides. Sections were deparaffinized and rehydrated through standard procedures.

Sections were blocked for 1 h at room temperature in a humid chamber using phosphate-buffered saline (PBS; pH 7.4) containing 0.05% Tween-20 and 5% non-fat milk. After washing three times with PBS-Tween (0.05%), slides were incubated overnight at 4°C with each of the 18 monoclonal antibodies.Following three additional washes, sections were incubated for 1 h at room temperature with a DyLight^™^ 488–conjugated goat anti-mouse secondary antibody (anti-IgG + anti-IgM; KPL). Secondary antibody–only controls were included by replacing the primary antibody with PBS while maintaining incubation with the secondary antibody under identical conditions. Slides were then washed, mounted with buffered glycerin, and examined under a Carl Zeiss epifluorescence microscope. Images were captured using a Canon PowerShot A3300 IS digital camera (8.2 megapixels).

Although this approach allowed antigen detection in parasite tissue, calcareous corpuscles were frequently detached during tissue processing and staining, limiting reliable evaluation of monoclonal antibody reactivity toward corpuscle-associated antigens.

#### 2.7.2 Immunofluorescence in isolated calcareous corpuscles

To overcome the limitations observed in parasite tissue sections, immunofluorescence assays were subsequently performed using isolated calcareous corpuscles fixed directly onto glass slides. Previously purified calcareous corpuscles were deposited by placing 15 μL of corpuscle suspension onto poly-L-lysine–coated glass slides and allowing the samples to air-dry at room temperature. Slides were then fixed in 70% ethanol for 20 min and washed with PBS.

Immunolocalization was carried out using the same immunofluorescence protocol described for parasite tissue sections. All 18 monoclonal antibodies were evaluated individually as primary antibodies, followed by incubation with DyLight^™^ 488–conjugated anti-mouse secondary antibody (anti-IgG + anti-IgM). Fluorescence signals were visualized using a Carl Zeiss immunofluorescence microscope, and images were recorded with a Canon PowerShot A3300 IS digital camera.

## 3. Results

### 3.1 Standardization and assessment of calcareous corpuscle isolation

The study procedures comprised: (i) extraction and purification of calcareous corpuscles, and (ii) controlled dissolution to obtain corpuscle-associated proteins.

**Figure 1:**
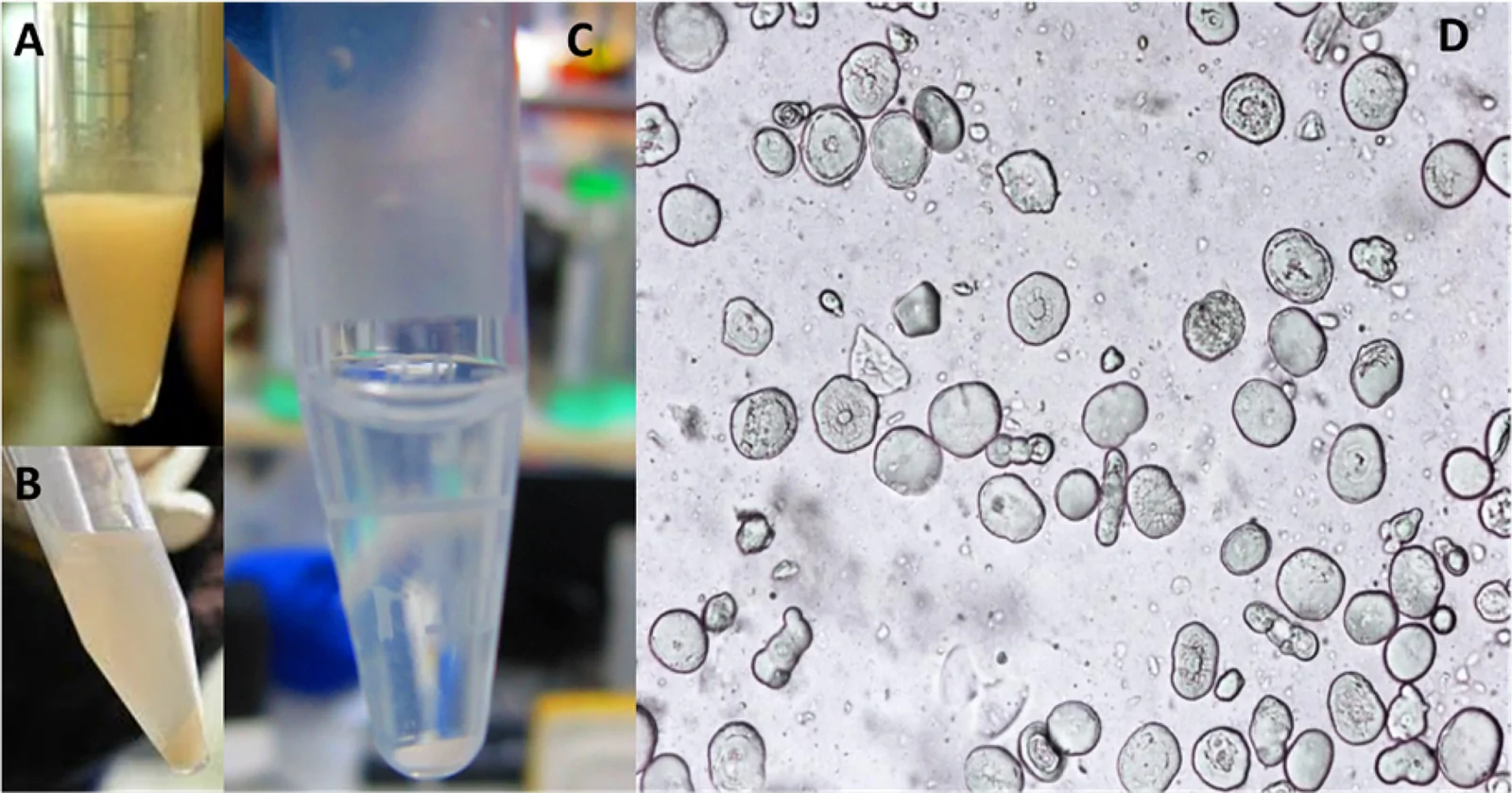
Isolation of calcareous corpuscles from *T. solium*. (a, b and c) Formation of a reproducible whitish pellet indicates enrichment of calcareous corpuscles. (d) Light microscopy image of calcareous corpuscles observed at 400× magnification, showing characteristic spherical morphology.

The extraction phase involved mechanical disruption followed by successive washing and differential centrifugation steps until obtaining a clean sediment devoid of visible cellular debris (Fig. 1). A whitish pellet corresponding to corpuscle-enriched preparation was obtained across repeated preparations (Fig. 1a–c).

Microscopic examination confirmed the presence of spherical calcareous corpuscles displaying the characteristic concentric morphology (Fig. 1d), indicating preservation of their structural integrity after isolation.

Following isolation, calcareous corpuscles were subjected to controlled dissolution using sulfamic acid solution. A total of fifteen sequential supernatants were collected throughout the dissolution process. Progressive structural disruption of the corpuscles was observed, and by the fifteenth fraction, no intact calcareous corpuscles were detectable under light microscopy, indicating complete dissolution of the mineral matrix.

The resulting supernatants contained soluble material recovered during sequential corpuscle dissolution and were subsequently used for protein quantification and electrophoretic characterization.

### 3.2 Protein quantification of dissolved calcareous corpuscles

Protein concentration was determined in duplicate for the fifteen sequential supernatants obtained during sulfamic acid dissolution of calcareous corpuscles. Quantification was performed using the Bradford method, and concentrations were calculated based on a bovine serum albumin (BSA) calibration curve (see Supplementary Fig. S1).

Protein concentrations varied among fractions, ranging from 23.83 to 161.07 μg/mL (see Supplementary Table S2). Notably, supernatants 8 and 9 exhibited the highest protein concentrations, reaching 161.07 μg/mL and 103.21 μg/mL, respectively. These fractions represented the peak of protein release during the dissolution process.

The observed pattern is compatible with a non-uniform release of corpuscle-associated proteins, with a marked increase in protein recovery in the intermediate dissolution fractions, followed by a gradual decrease toward the final fractions.

Based on these results, supernatants 8 and 9 were selected for subsequent electrophoretic characterization using the highest protein concentrations obtained.

### 3.3 Electrophoretic profile of calcareous corpuscle proteins

Aliquots from supernatants 8 and 9 were analyzed by SDS-PAGE to evaluate the protein composition of dissolved calcareous corpuscles at their maximal recovered concentrations. In addition, supernatant 1 was analyzed to determine whether protein bands could already be detected in the initial stages of corpuscle dissolution (Fig. 3).

**Figure 2:**
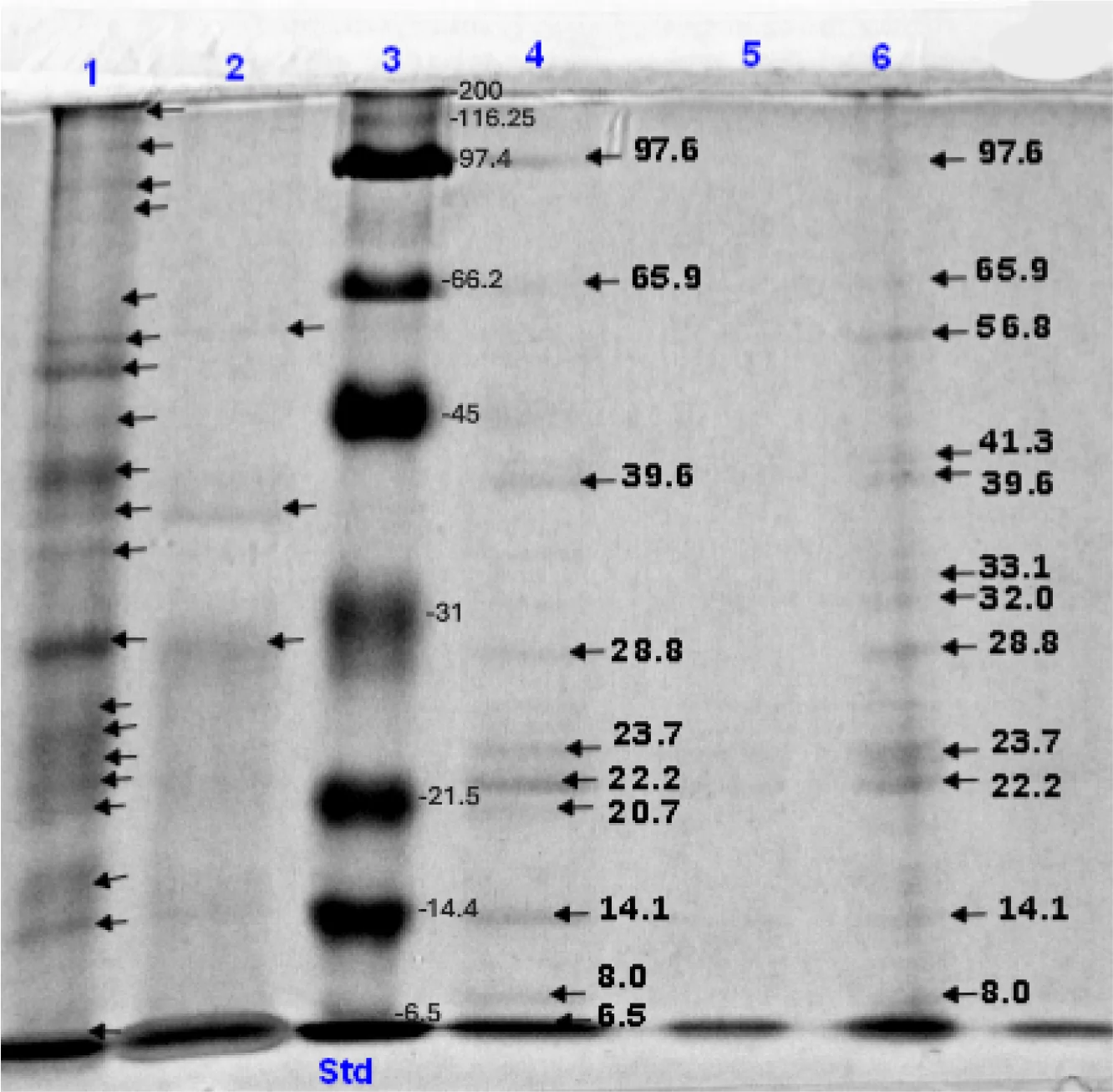
SDS-PAGE profile of calcareous corpuscle proteins. Electrophoretic analysis of proteins obtained from dissolved calcareous corpuscles of *T. solium*. Lane 1: whole cysticercus antigens; Lane 2: cysticercus vesicular fluid antigens; Lane 3: molecular weight marker; Lane 4: supernatant 8; Lane 5: supernatant 1; Lane 6: supernatant 9. Proteins were separated by SDS-PAGE and visualized by silver staining.

Supernatant 8 displayed the most complex electrophoretic profile, with fourteen distinct protein bands corresponding to approximate molecular weights of 98.1, 66.9, 44.7, 39.9, 34.6, 31.8, 28.8, 25.8, 23.7, 22.2, 20.7, 14.1, 8.0, and 6.5 kDa. Supernatant 9 showed a similar banding pattern, including proteins of 66.9, 39.9, 34.6, 31.8, 28.8, 25.8, 23.7, 22.2, 14.1, 8.0, and 6.5 kDa. In addition, this sample revealed nine additional bands with estimated molecular weights of 97.9, 94.2, 56.8, 43.9, 41.3, 33.1, 19.3, 16.5, and 15.6 kDa (Fig. 2). Supernatant 1 and supernatants 13, 14, and 15 (not shown in the figure), exhibited fewer and less intense bands, consistent with their lower protein concentrations determined by the Bradford assay. Overall, a total of twenty-three distinct protein bands were identified across all analyzed fractions, indicating a heterogeneous protein composition associated with calcareous corpuscles.

Based on these results, supernatants 8 and 9 were pooled and subsequently used for electrotransfer onto nitrocellulose membranes and immunodetection with monoclonal antibodies.

### 3.4 Immunodetection and selection of monoclonal antibodies recognizing calcareous corpuscle-associated antigens

**Figure 3:**
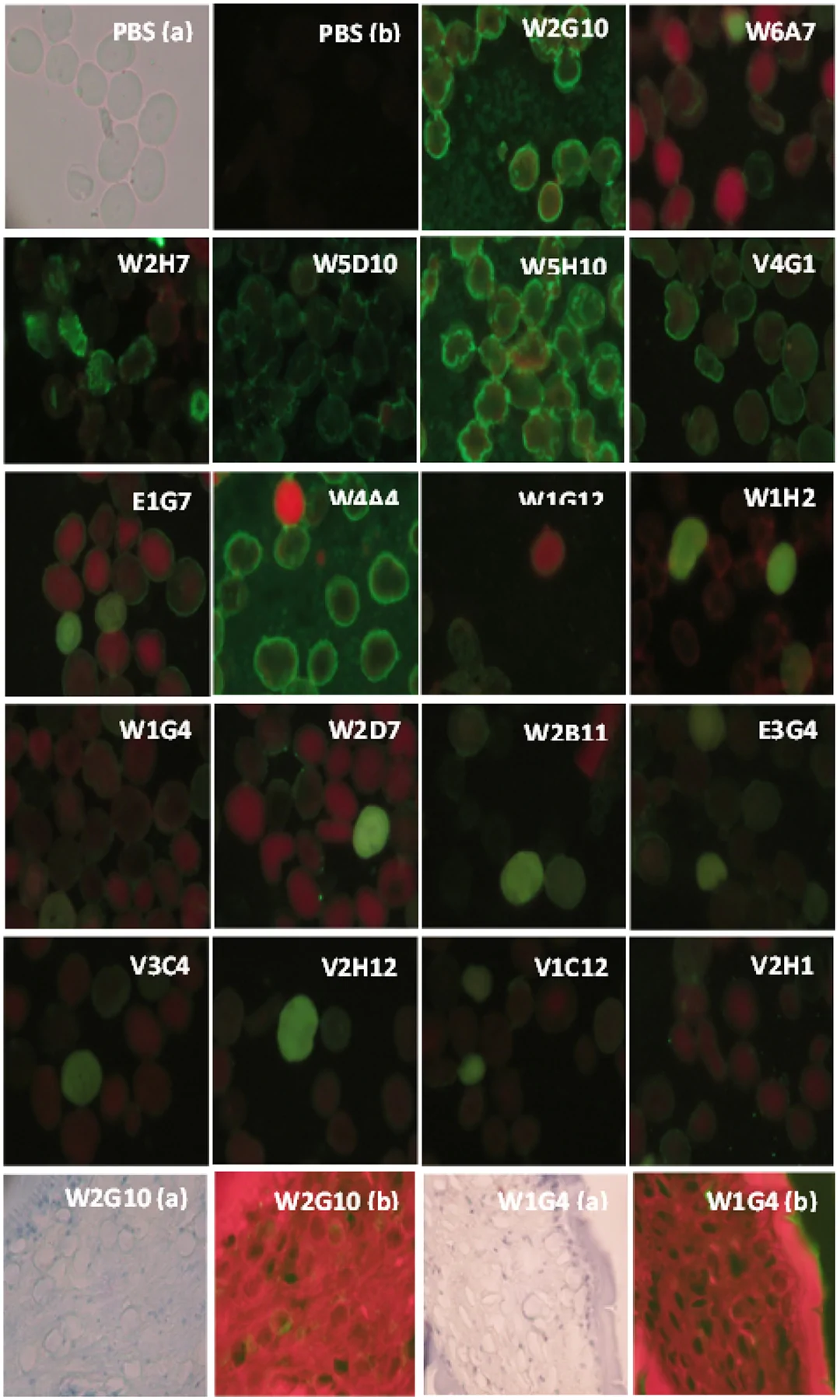
Immunofluorescence screening of monoclonal antibodies on isolated calcareous corpuscles and comparison with parasite tissue sections. Immunofluorescence analysis of isolated calcareous corpuscles from *T. solium* fixed on glass slides using a panel of monoclonal antibodies. PBS-treated corpuscles (secondary antibody–only control) were used as negative controls and are shown under visible light (PBS a) and fluorescence microscopy (PBS b). Positive immunofluorescent reactions were observed with monoclonal antibodies W2G10, W6A7, W2H7, W5D10, W5H10, V4G1, W4A4, and E1G7, showing specific DyLight-associated fluorescence localized to calcareous corpuscles. Negative reactions were observed with monoclonal antibodies W1G12, W1H2, W1G4, W2D7, W2B11, E3G4, V3C4, V2H12, V1C12, and V2H1, showing only background signal or intrinsic autofluorescence. The lower panels show immunofluorescence results obtained using parasite tissue sections. Representative images for W2G10 (positive) and W1G4 (negative) are shown. All images were acquired at 400× magnification, except tissue section images (W2G10 a–b and W1G4 a–b), which were acquired at 200× magnification. Specific DyLight-associated fluorescence was distinguishable from the weak and diffuse intrinsic autofluorescence of calcareous corpuscles.

**Figure 4:**
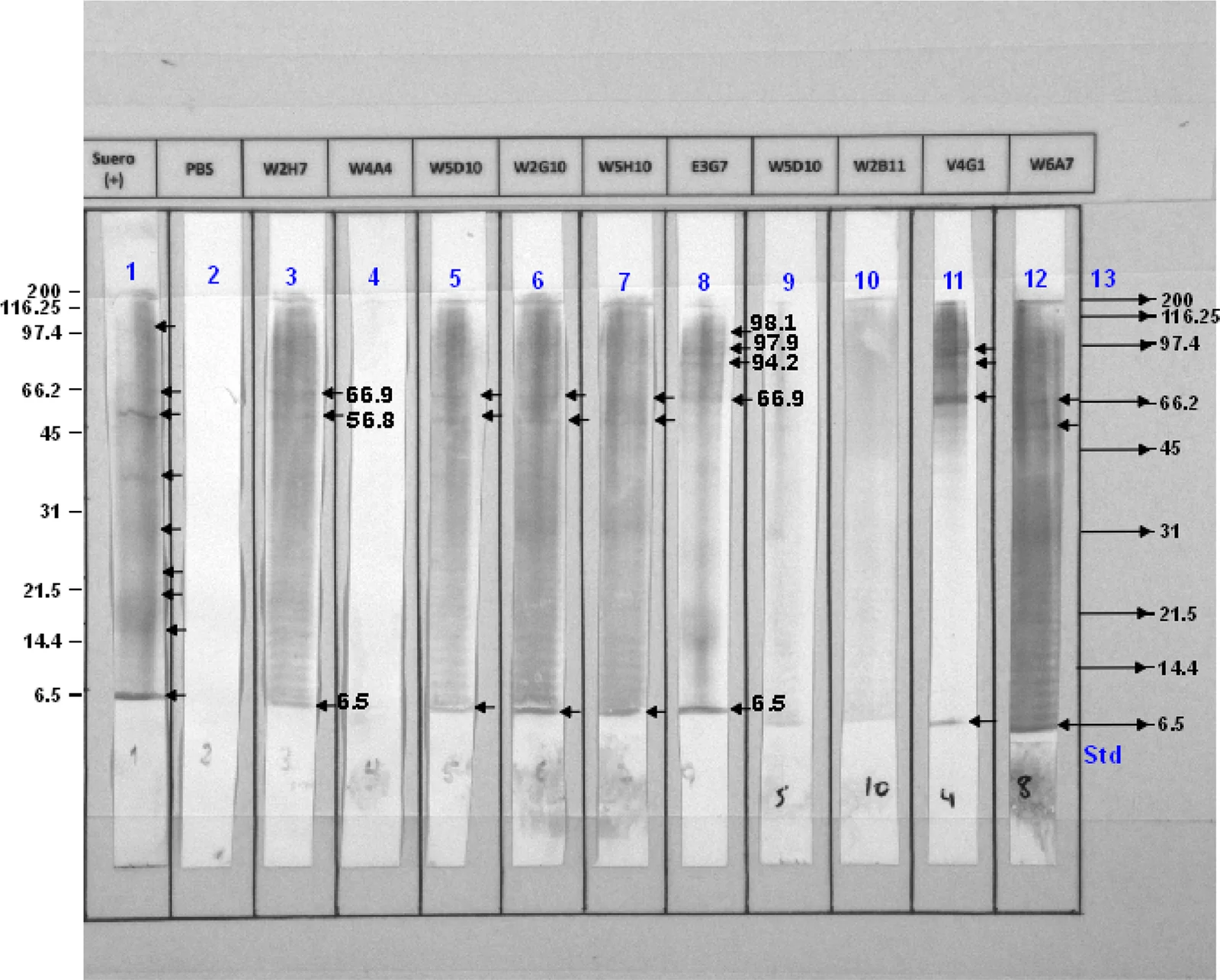
Western blot analysis of calcareous corpuscle-associated antigens using monoclonal antibodies against *T. solium*. Western blot immunodetection of proteins extracted from calcareous corpuscles using selected monoclonal antibodies. Lane 1: positive control (serum from mice immunized with whole *T. solium*cysticercus antigen); Lane 2: negative control (PBS); Lane 3: W2H7; Lane 4: W4A4; Lane 5: W5D10; Lane 6: W2G10; Lane 7: W5H10; Lane 8: E1G7; Lane 9: V2H12; Lane 10: W2B11; Lane 11: V4G1; Lane 12: W6A7.

Immunolocalization assays were performed using the same monoclonal antibody panel, in order to detect calcareous corpuscle-associated antigens by immunofluorescence. Initial trials on parasite tissue sections were unsuccessful because calcareous corpuscles present within the sections were frequently detached from the tissue matrix. Immunofluorescence assays were subsequently performed using isolated calcareous corpuscles fixed directly onto glass slides. This approach preserved corpuscle integrity and enabled improved visualization of antibody binding (Fig. 3). Isolated corpuscles were incubated with individual monoclonal antibodies followed by DyLight-conjugated anti-mouse secondary antibodies. Using this approach, eight monoclonal antibodies exhibited clear positive immunofluorescent reactivity with calcareous corpuscles: W2G10, W6A7, W2H7, W5D10, W5H10, V4G1, E1G7, and W4A4. Ten monoclonal antibodies showed no detectable reactivity, including W1G12, W2D7, W1H2, W1G4, V3C4, V2H12, V1C12, V2H1, W2B11, and E3G4 (Fig. 4). Although calcareous corpuscles displayed a degree of intrinsic autofluorescence, this signal was clearly distinguishable from the specific DyLight-associated fluorescence, which was brighter, localized, and antibody-dependent.

Based on these results, ten monoclonal antibodies were selected for subsequent Western blot analysis. This group included all antibodies showing positive reactivity (W2G10, W6A7, W2H7, W5D10, W5H10, V4G1, E1G7, and W4A4) and two antibodies with negative immunofluorescence reactivity (W2B11 and V2H12) selected at random as specificity controls. This selection strategy enabled comprehensive evaluation of calcareous corpuscle-associated proteins while allowing discrimination between specific and nonspecific antibody recognition in subsequent immunoblot assays.

### 3.5 Western blot analysis of calcareous corpuscle-associated proteins

Western blot analysis was performed using the monoclonal antibodies selected based on their immunofluorescence reactivity against isolated calcareous corpuscles. Protein extracts obtained from pooled supernatants of dissolved calcareous corpuscles were separated by SDS-PAGE and transferred onto nitrocellulose membranes for immunodetection (Fig. 4). As a positive control, serum from mice immunized with whole *T. solium* cysticercus antigen was included, while PBS was used as a negative control.

The monoclonal antibodies previously classified as positive by immunofluorescence on calcareous corpuscles fixed on glass slides recognized protein bands in Western blot assays (Fig. 4). In contrast, monoclonal antibodies selected as negative controls, and negative by immunofluorescence, did not recognize any bands under the same experimental conditions.

Monoclonal antibodies W2H7, W5D10, W2G10, W5H10, and W6A7 consistently detected protein bands with estimated molecular weights of 66.9, 56.8, and 6.5 kDa. The monoclonal antibody E1G7 recognized multiple protein bands corresponding to 98.1, 97.9, 94.2, 66.9, and 6.5 kDa. Similarly, monoclonal antibody V4G1 detected protein bands at 97.9, 94.2, 66.9, and 6.5 kDa, showing particularly strong reactivity toward the 66.9 kDa band (Fig. 4).

In contrast, monoclonal antibodies W4A4, V2H12, and W2B11 did not recognize any protein bands in Western blot assays, indicating lack of detectable reactivity under the conditions tested (Fig. 4).

Overall, these results demonstrate that a defined subset of monoclonal antibodies recognizes a specific group of calcareous corpuscle-associated proteins, with recurrent detection of proteins in the ~ 66.9 kDa and low-molecular-weight ranges.

## 4. Discussion

Despite being a ubiquitous component of cestodes, the molecular composition and antigenic properties of calcareous corpuscles remain poorly characterized, particularly in Taenia solium. This study provides both methodological and immunological contributions to the understanding of these structures. By developing a reproducible two-phase approach for corpuscle isolation and controlled dissolution, we were able to recover and characterize a diverse set of corpuscle-associated proteins. The findings support the view that calcareous corpuscles are not inert mineral deposits but rather complex organo-mineral structures containing immunologically relevant proteins. This work expands current knowledge of calcareous corpuscles and provides a practical framework for future proteomic, immunological, and diagnostic studies.

Here we developed and validated a reproducible two-phase methodology that enables the isolation of intact calcareous corpuscles from viable *T. solium* cysticerci and the subsequent recovery of their associated proteins, allowing the recovery of a broader spectrum of corpuscle-associated proteins, without restricting the analysis to calcium-affinity components, thereby expanding the molecular characterization of these structures.

The use of viable cysticerci obtained from naturally infected pigs, combined with controlled mechanical homogenization and differential centrifugation, consistently yielded morphologically intact calcareous corpuscles. Microscopic validation confirmed preservation of the characteristic spherical and concentric architecture prior to dissolution, supporting the specificity and robustness of the isolation procedure. This step is critical, as contamination with surrounding parasite tissue or premature structural disruption may compromise downstream biochemical analyses.

Controlled dissolution of calcareous corpuscles using sulfamic acid enabled progressive release of embedded proteins, with peak protein recovery observed in intermediate fractions. This pattern suggests that proteins are differentially distributed within the mineralized matrix rather than being superficially adsorbed, supporting the concept that calcareous corpuscles are complex organo-mineral assemblies (von Brand and Nylen, 1970; Vargas-Parada and Laclette, 1999; Yang, 2000; Park et al., 2005). Complete dissolution was confirmed microscopically, providing direct visual validation of protein release before biochemical and immunological characterization.

Electrophoretic analysis revealed a heterogeneous protein profile comprising at least 23 distinct protein bands associated with calcareous corpuscles. Several of these proteins fall within molecular weight ranges previously reported for *T. solium* antigens, most notably the recurrent detection of a ~ 66.9 kDa protein. Proteins in this molecular weight range have been described in cysticercus extracts and shown to be immunogenic in both experimental models and human infection (Tsang, Brand and Boyer, 1989; Rodriguez-Canul et al., 1997; Park et al., 2000; Barcelos et al., 2001; Shukla et al., 2008; Romo et al., 2020). The consistent recognition of this protein by multiple monoclonal antibodies in the present study suggests that it represents a conserved and immunologically relevant component of calcareous corpuscles.

The immunological relevance of corpuscle-associated proteins was further supported by Western blot analysis using monoclonal antibodies generated against different *T. solium* antigenic preparations. As expected, monoclonal antibodies raised against whole cysticercus extracts (TsW) recognized calcareous corpuscle-associated proteins, since these antigens are present within the total parasite extract used for immunization (Paredes et al., 2016). This observation confirms that calcareous corpuscles share antigenic components with other structural compartments of the parasite.

Importantly, monoclonal antibodies generated against vesicular fluid antigens (TsV) also recognized multiple calcareous corpuscle-associated proteins. In particular, monoclonal antibody V4G1 detected proteins of approximately 97.9, 94.2, 66.9, and 6.5 kDa, suggesting that these molecules are present both in vesicular fluid and in calcareous corpuscles. This finding points to the existence of shared antigen pools or molecular trafficking processes (von Brand et al., 1960, 1967; McCullough and Fairweather, 1987; Vargas-Parada and Laclette, 1999) between vesicular fluid and mineralized structures. The strong reactivity of V4G1 toward the 66.9 kDa and 6.5 kDa proteins is consistent with the potential antigenic relevance.

Similarly, monoclonal antibodies generated against excretion–secretion antigens (TsE) recognized calcareous corpuscle-associated proteins. The monoclonal antibody E1G7 detected proteins of approximately 98.1, 97.9, 94.2, 66.9, and 6.5 kDa, indicating that these molecules are not only structurally associated with calcareous corpuscles but are also physiologically released into the parasite’s external environment. This observation is consistent with previous secretome studies, which have described overlapping antigenic components between secreted products and internal parasite compartments (Gomez et al., 2015; Vega-Angeles et al., 2019; Arora et al., 2023; Rawat et al., 2024).

In addition to immunoreactive proteins, several calcareous corpuscle-associated proteins detected by SDS-PAGE were not recognized by the monoclonal antibodies evaluated in this study. These proteins, spanning molecular weights from approximately 8 to 43.9 kDa, may represent structural or metabolic components of calcareous corpuscles that are weakly immunogenic or not readily exposed to the host immune system.

From a methodological perspective, the use of isolated calcareous corpuscles fixed on glass slides for immunofluorescence proved superior to parasite tissue sections, in which corpuscle loss during histological processing limited reliable antibody discrimination. This approach enabled accurate screening of monoclonal antibody reactivity and may serve as a useful platform for future antigen localization and antibody selection studies in *T. solium* and other helminths.

Our study presents some limitations that should be considered when interpreting the results. Protein characterization was based on electrophoretic migration and immunoreactivity patterns, and definitive protein identification was not performed. Proteomic approaches such as mass spectrometry will be required to confirm the molecular identity of the calcareous corpuscle-associated proteins detected in this study. Although a diverse panel of monoclonal antibodies was employed, these antibodies were generated against whole cysticercus extract, vesicular fluid, or excretion–secretion antigens rather than calcareous corpuscle-specific proteins. Consequently, some corpuscle-associated proteins may not have been recognized due to limited antigen exposure or low immunogenicity. Immunofluorescence-based screening relied on isolated calcareous corpuscles fixed on glass slides rather than in situ localization within intact parasite tissue. While this approach improved antibody discrimination and prevented loss of corpuscles during histological processing, it does not fully reflect the spatial organization of calcareous corpuscles within the parasite. Finally, corpuscle enrichment was supported primarily by morphological criteria and repeated washing steps rather than by orthogonal purity markers; therefore, residual contamination from surrounding parasite material cannot be fully excluded. Likewise, sequential sulfamic acid dissolution may preferentially recover acid-stable proteins and may alter labile proteins or epitopes, potentially biasing the antigenic profile observed in the recovered fractions.

## 5. Conclusion

A reproducible two-phase methodology was developed for the isolation of calcareous corpuscles from viable *T. solium* cysticerci and for the recovery of their associated proteins. This approach enabled preservation of corpuscle integrity, complete dissolution of the mineral matrix, and effective release of corpuscle-associated proteins.

Calcareous corpuscles were shown to contain a diverse set of immunoreactive proteins, several of which are shared with other parasite compartments, including vesicular fluid and excretion–secretion products. The identification of recurrent protein bands, particularly around 66.9 kDa and in the low-molecular-weight range, supports the antigenic relevance of these structures.

Overall, this study provides methodological and experimental evidence that calcareous corpuscles are complex organo-mineral structures rather than inert deposits and establishes a practical framework for future proteomic and immunological studies in *T. solium*.

### Supplementary Material

Supplementary Files

This is a list of supplementary files associated with this preprint. Click to download.

- PROTEINASCCrecortadosconPM2026ultimo.png
- westernblotCC.tif
- SupplementaryTableSandFigures.docx

## Data Availability

All data generated or analyzed during this study are included in this published article and its supplementary information files.
